# Notes of the Week

**Published:** 1921-08-13

**Authors:** 


### NOTES OF THE WEEK.

### PLANS AND PROSPECTS AT CARDIFF.

In regard to the action following the report of the Cave Committee, the committee of King Edward VII. Hospital, Cardiff, of which Sir W. H. Diamond is chairman, has been quick to recognise that little can be hoped from the Government grant because of its limited amount and the number of applicants. The finance committee will issue its plans to the public soon after the holidays, and in the meantime two suggestions are made to every reader of Sir W. Diamond's letter to the Cardiff newspapers. The first is for the reader to send a subscription if he has not done so already; the second, which can best be done when the former suggestion has been adopted, is to send the name and address of any colliery, workshop, office, or place of employment where the reader knows that no weekly collection is held. The information will be followed by a visit or a letter, probably both. Sir William Diamond well remarks that the spontaneous help which is here invited has a great moral value "in example and encouragement." When it is remembered that the revenue from weekly contributions is only about half what it might be in the district, we hope that many letters will be received from friends of the hospital stating that in "my" or "our" place of employment there is no collection at present. The initiative coming from the men themselves would be the best encouragement of all, and those men who already subscribe are in the best position to mention places at present untouched by the movement. It will also be interesting to learn what the new local hospital committee for the area proposes for the Cardiff hospitals, for which some common policy will be decided.

### REGIONAL COMMITTEES AND NEW HOSPITALS.

The various degrees of success which have attended war memorial schemes for the building of new hospitals make us regret that the Regional Committees of the British Hospitals Association were not in existence earlier. For example, at North Walsham a cottage hospital was decided on, and a sum of over £6,000 has been collected, divided between the endowment and the building funds. But some have suggested that the money collected would be better spent if handed to the Norfolk and Norwich Hospital, a view that is understandably criticised by the supporters of the North Walsham scheme. Now, had a Regional Committee of the British Hospitals Association been in existence at the time when this form of memorial was adopted the scheme might have had the backing of all the hospitals of the area, or at worst met with a serious obstacle at the very start. Will anyone suggest that because the Regional Committees are composed of men associated with existing hospitals that no scheme for new hospitals would be encouraged? The present is a simple instance of the lack of co-ordination in hospital affairs and of the divided counsels which make intelligent progress difficult. Mr. J. Dixon and Dr. C. H. W. Page, who have argued strongly in favour of the scheme, would, no doubt, welcome an expression of opinion from the recently formed Regional Committee.

### A MISUNDERSTANDING AT BRIGHTON.

One of the remarkably few instances in which patients' payments have threatened to interfere with weekly contributions comes from Brighton, where some workpeople are hesitating to continue the latter because patients are expected to give what they can afford. In a judicious statement, Miss Mary Cole, the hospital's appeal secretary, reminds these contributors that even with patients' payments the hospital needs to collect £25,000 a year, and, if voluntary support is withheld, will have to reduce its beds. The hesitation is the more remarkable because the Royal Sussex County Hospital has now become famous through the original idea of its Sussex scheme, which three well-known London hospitals are copying and other institutions all over the country are watching with great interest. Consequently, we should have thought that all the hospital's friends would have been unusually proud of their institution at this moment, and in their desire for it to retain the leadership which it has won would have made exceptional efforts to finance it.

### ANNUAL MEETINGS AWAY FROM HOME.

Earlier in the year the Sleaford Hospital Saturday Committee wrote to the board of management of Lincoln County Hospital to suggest that the annual meeting of the hospital should be held at Sleaford. At a special meeting of the former, presided over by Mr. N. E. Snow, it was announced that the hospital and the Lincoln Branch Committee have agreed, and think that to hold the annual meeting at Sleaford will help to keep the hospital movement before the county. The details can be arranged easily. The meeting will take place later in the year at the Town Hall, where cups, medals and diplomas will be presented, and a tea will probably be given at the Church Hall afterwards. The local collections are being organised upon the spot, and if the hospital is to be placed upon a sound footing it is essential that the support from the county should be increased. The experiment will be watched with interest, and its origin in a local suggestion may lead other county hospitals to consider adopting a similar plan. If found successful, is there any reason why the annual meeting should not be held in a different place each year? It might be held in any county centre which thought the plan would be beneficial.

### THE DOCTORS AND ST. LUKE'S HOSPITAL.

At the request of the Ministry of Health, the Bradford Health Committee has been in long negotiation with the medical advisory committee of the city in respect of a scheme to satisfy the profession in regard to St. Luke's, the Municipal General Hospital. The Health Committee now reports that satisfactory conclusions have been formed, and it has therefore decided to advertise for candidates to fill the medical posts at the hospital.

### NO PAYING PATIENTS AT CAMBERWELL INFIRMARY.

Camberwell Board of Guardians have abandoned for the present their proposal that a ward should be set aside for paying patients. An appeal was made to the Ministry of Health for sanction to the scheme, and the reply was far from encouraging, the Ministry stating that the Minister was not able by any alteration of the Orders in force to empower the guardians to give assistance to cases which they are not at present legally empowered to relieve, or to vary the statutory provisions affecting the recovery of the cost of relief. This means that the guardians cannot say to a patient, "Come inside; we will look after you if you pay us three guineas a week." But if the patient cannot obtain admittance to a hospital and requires medical and surgical treatment which cannot be obtained at home, then the patient is destitute. She or he will be admitted to the guardians' infirmary, and the full cost of the patient's maintenance, even if it is three guineas a week, can legally be claimed from the relatives. Wherein lies the difference between these two types only the Ministry of Health can say.

### THE SCHOOL-TEACHER'S OPPORTUNITY.

The East Lancashire Workpeople's Hospital Fund, which has lately made excellent progress, is trying to secure a uniform effort, not only in the factories, but in the trades, businesses, and professions. Its secretary, Mr. Herbert Holden, lately addressed the Blackburn and District Teachers' Association. There is, of course, a double reason why their co-operation would be of value. Apart from the immediate benefit of any school collections which the teachers might be interested to begin, the habit of giving to hospitals is best formed, like other good habits, in childhood; and a body of public opinion already interested in the maintenance of voluntary hospitals should go out from the schools every year into every walk of life. Only by such a stream of young recruits can the uniformity asked for by Mr. Holden be secured. The real maintenance of the voluntary system is the educated public opinion behind it. The children taught in the schools can learn all professions. The teachers, no doubt, realise this, and are aware that the proper organisation of school collections does not end with the counting of pennies. It includes a plan for bringing the school children into personal touch with their local hospital, which they should visit, and about which they should hear. The periodical presentation of the proceeds should be made the occasion of a function to bring the hospital and the children together. Such a relation can be educational in the best sense, and, in the long run, more depends upon the co-operation of the teachers than on any other body which Mr. Holden may address.

### FORESTERS AND PANEL DOCTORS.

The Chief Ranger, Mr. H. G. Hutchins, speaking at the High Court of the Ancient Order of Foresters' Friendly Society, strongly criticised the Health Insurance Act. He expressed the opinion that the Act, which was brought into being to succour the weak in their time of need, has only resulted in benefiting the doctors. Some of the evils associated with the system would be remedied if every member of the medical profession appreciated his obligation to insured patients, and to ensure this it would seem to be necessary considerably to curtail the number of panel patients even now allotted to panel practitioners. Nothing was said at the High Court about devoting any part of the surplus funds to the hospitals, without which, it is now generally admitted, the National Health Scheme would have proved unworkable. There was, however, an indication that it is the policy of the Society to utilise some portion of future disposable surpluses in the direction of preventing sickness and disease.

### PENSIONS FOR SEAMEN.

It is not generally known that there is a fund in connection with the National Health Insurance Acts which provides pensions for masters, seamen, and apprentices to the sea service or sea-fishing service who are members of approved societies under the Acts. The fund is commonly known as the "Lascar Fund," and its revenue is partly derived from Health Insurance contributions paid by shipowners in respect of seamen who neither are domiciled nor have a place of residence in the United Kingdom. There is a special governing body under the Chairmanship of Sir Norman Hill, Bart., and awards will be made, commencing on January 1, 1922, of 200 pensions of the value of 5s. a week each to persons, as described above, who are between the ages of sixty-five and seventy on that date. Other benefits may be awarded at the discretion of the governing body. Applications should be made to the Secretary, "Lascar Fund," the Ministry of Health, Insurance Department, Buckingham Gate, London, S.W. 1.

### THE MINERS' HALFPENNY AT WORKSOP.

The Worksop Victoria Hospital is in difficulties with a small but growing deficit. At the same time it derives some of its income from weekly contributions, but these, at least from the miners, amount only, we understand, to a halfpenny per week. Surely that is satisfactory to neither party. It has been decided to address an appeal to the workers, and we hope that this will include a reminder that a halfpenny per week everywhere else is regarded as too little even for children. The new weekly subscribers whom it is hoped to attract will surely begin at least with one penny.

